# Two Times Versus Four Times Daily Cephalexin Dosing for the Treatment of Uncomplicated Urinary Tract Infections in Females

**DOI:** 10.1093/ofid/ofad430

**Published:** 2023-08-11

**Authors:** Aidan Yetsko, Heather M Draper, Kristen Eid, Andrew P Jameson, Lisa E Dumkow

**Affiliations:** Department of Pharmacy, Trinity Health Grand Rapids Hospital, Grand Rapids, Michigan, USA; Department of Pharmacy, Trinity Health Grand Rapids Hospital, Grand Rapids, Michigan, USA; Department of Pharmacy, Trinity Health Grand Rapids Hospital, Grand Rapids, Michigan, USA; Division of Infectious Disease, Trinity Health Grand Rapids Hospital, Grand Rapids, Michigan, USA; College of Human Medicine, Michigan State University, Grand Rapids, Michigan, USA; Department of Pharmacy, Trinity Health Grand Rapids Hospital, Grand Rapids, Michigan, USA; Division of Infectious Disease, Trinity Health Grand Rapids Hospital, Grand Rapids, Michigan, USA

**Keywords:** adherence, cephalexin, dosing, optimization, urinary tract infection

## Abstract

**Background:**

The current treatment guidelines of the Infectious Diseases Society of America recommend β-lactam antibiotics as alternative rather than first-line agents for the treatment of uncomplicated urinary tract infection (uUTI). Cephalexin is a commonly prescribed first-generation cephalosporin with excellent bioavailability and urinary penetration; however, little data exist to support optimal dosing for uUTI.

**Methods:**

This retrospective multicenter cohort study included adult female patients who received 5 to 7 days of cephalexin for symptomatic uUTI with a cefazolin-susceptible urine culture. The primary objective was to compare uUTI treatment failure (eg, continued or recurrent symptoms within 30 days) between patients treated with cephalexin 500 mg twice daily (BID group) and 500 mg 4 times daily (QID group) in the outpatient setting. Secondary outcomes included time to treatment failure, reported adverse events within 7 days of treatment, and occurrence of *Clostridioides difficile* within 30 days of treatment.

**Results:**

A total of 261 patients were included (BID, n = 173; QID, n = 88). Baseline characteristics were similar between the groups. *Escherichia coli* was the most commonly isolated pathogen (85.4%). There was no difference in treatment failure observed between the groups (BID 12.7% vs QID 17%, *P* = .343), including failure while undergoing therapy (BID 2.3% vs QID 5.7%, *P* = .438) or recurrence within 30 days (BID 10.4% vs QID 11.3%, *P* = .438). No differences in reported adverse events (BID 4.6% vs QID 5.6%, *P* = .103) were observed between groups.

**Conclusions:**

Twice-daily cephalexin is as effective as 4-times-daily dosing for uUTI. A twice-daily dosing strategy may improve patient adherence.

## Teaser

Cephalexin dosed 500 mg twice daily was equally safe and effective as 4-times-daily dosing in the treatment of female patients with uncomplicated urinary tract infections. Less frequent dosing may improve patient adherence and satisfaction with treatment.

## Graphical Abstract

**Graphical Abstract:**
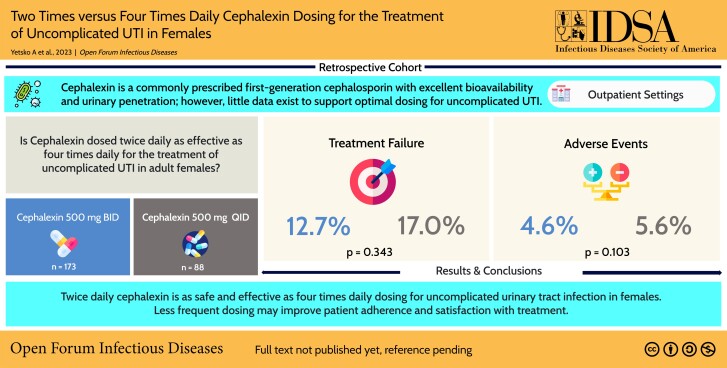
https://tidbitapp.io/tidbits/two-times-versus-four-times-daily-cephalexin-dosing-for-the-treatment-of-acute-cystitis-in-females

Urinary tract infections (UTIs) are one of the most common indications for outpatient antibiotic prescribing, with millions of Americans treated for UTI annually [1]. In 2011, the Infectious Diseases Society of America published national best practice recommendations to guide the treatment of uncomplicated UTI (uUTI), which recommended nitrofurantoin, trimethoprim-sulfamethoxazole (TMP/SMX), or fosfomycin as first-line empiric therapies [2]. Alternative oral antimicrobials, including fluoroquinolones or β-lactams, were recommended to be reserved for patients who could not receive a first-line agent due to allergy history, comorbidities, susceptibility, tolerance, or availability [2]. In the decade following the publication of these guidelines, antimicrobial resistance and increasing patient complexity have continued to challenge the way that uUTIs are treated [3]. In the United States between 2011 and 2019, rates of urinary *Escherichia coli* isolates showing resistance to TMP/SMX and fluoroquinolones were 25.4% and 21.1%, respectively [4]. Additionally, serious adverse drug reactions, medication interactions, and comorbidities, such as acute or chronic renal insufficiency, may limit the use of first-line uUTI agents and fluoroquinolones [5, 6].

Cephalexin, an oral first-generation cephalosporin, has a bioavailability of 90%, demonstrates low protein binding, and achieves high urinary concentrations, making it an ideal choice for uUTI treatment [7, 8]. Despite cephalexin's long history of clinical utility in treating common infections in the outpatient setting, limited data exist to support the optimal dosing of cephalexin for uUTI. Common references recommend 250 to 500 mg dosed every 6 hours; however, pharmacokinetic data indicate that a 12-hour dosing frequency may be adequate [8, 9]. As decreasing medication dosing frequency has shown to improve patient adherence and treatment success, the purpose of this study was to compare clinical outcomes between adult female patients treated for uUTI with cephalexin 500 mg twice daily and 500 mg 4 times daily in the outpatient setting [10, 11].

## METHODS

### Study Design and Population

This retrospective cohort study evaluated adult patients treated for uUTI in an outpatient setting across Trinity Health Michigan locations between 1 February 2020 and 31 August 2022. Outpatient setting was defined as antibiotic treatment received at discharge from an emergency department, urgent care site, or medical group office. Patients were eligible for study inclusion if they were females aged ≥18 years; had a diagnosis of acute cystitis or uUTI, with documented symptoms of dysuria, suprapubic pain, urinary frequency, or urinary urgency; had a urine culture with an organism isolated that was susceptible to cefazolin; and received a cephalexin prescription written for 5 to 7 days. Isolates were determined to be susceptible to cefazolin if they had a minimum inhibitory concentration (MIC) ≤16 mcg/mL. Cefazolin susceptibility serves as a surrogate for cephalexin susceptibility; cefazolin susceptibility was determined by automated methods with the Vitek 2 System (bioMerieux) [12]. Patients were excluded who were pregnant, had an indwelling urinary catheter or documented intermittent straight catheter, were diagnosed with pyelonephritis or complicated UTI, had systemic symptoms (eg, fever, chills, rigors), had a history of kidney transplant, were diagnosed with a recurrent UTI or had been treated for UTI within the previous 30 days, were currently prescribed chronic antibiotic prophylaxis, or were being treated for a concurrent infection. Patients were additionally excluded if they had no culture obtained or had a negative urine culture result; if ≥1 doses of intravenous (IV) antibiotics were administered prior to oral therapy; or if they had renal impairment with a creatinine clearance <30 mL/min, where dose-adjusted cephalexin would have been indicated.

### Patient Consent Statement

This study qualified for exemption after institutional review board evaluation and did not require patient consent.

### Data Collection

A report was generated from the electronic medical record (Epic Systems) of patients receiving cephalexin 500 mg 2 or 4 times daily for 5 to 7 days per the *ICD-10* diagnosis code for UTI (N39.0). Patients eligible for screening were then put into a randomized order and screened for inclusion until the desired sample size was met. Patient, treatment, and infection characteristics were collected from the medical record along with patient outcomes and follow-up characteristics recorded within 30 days of treatment completion.

### Study End Points

The primary objective of the study was to compare treatment failure between patients treated for uUTI in the outpatient setting with oral cephalexin 500 mg twice daily and 4 times daily. Treatment failure was defined as the combined endpoint of (1) continued symptoms or necessitation of new therapy while taking the initially prescribed 5 to 7 days of treatment and (2) treatment of recurrent symptomatic UTI within 30 days of therapy completion. Secondary objectives included comparing the time to failure between groups, retreatment characteristics, rate of patient-reported adverse events within 7 days of treatment completion, as well as *Clostridioides difficile* infection within 30 days of treatment completion. Adverse events were obtained from subsequent documentation in the electronic medical record and included nausea/vomiting, abdominal pain, diarrhea, headache, rash, hives, anaphylaxis, and vaginal yeast infection. Reviewed documentation included telephone encounters and follow-up evaluations with primary care or specialist providers, as well as subsequent encounters in the emergency department, urgent care center, or hospital.

### Statistical Analysis

Previously published literature assessing first-line therapies for uUTI have demonstrated that approximately 12% to 16% of uUTIs result in symptomatic recurrence necessitating retreatment within 28 days of initial therapy [13]. Based on this assumption, a sample size of 250 patients would be needed to detect at least a 10% absolute difference (14% vs 24%) in treatment failure between the groups according to a 2-sided test with an α value of .05 and power of 80%. A χ^2^ test or Fisher exact test was used to compare nominal data, as appropriate, and a Student *t* test or Mann-Whitney *U* test was used to compare interval data based on their distribution. Statistical analyses were performed with SPSS version 22 (IBM).

## RESULTS

**Figure 1.**
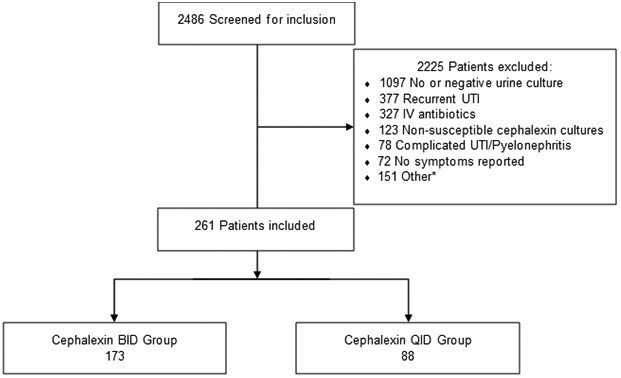
Patients evaluated for study inclusion. *Pregnancy, catheter use, creatinine clearance <30 mL/min, renal transplant, chronic antibiotics, or concurrent infection. BID, twice daily; IV, intravenous; QID, 4 times daily; UTI, urinary tract infection.

A total of 2486 patients were screened who were prescribed cephalexin for UTI with a duration of 5 to 7 days, with 261 meeting criteria for inclusion (Figure 1): 173 in the twice-daily (BID) group and 88 in the 4-times-daily (QID) group. Patient and treatment characteristics are outlined in Table 1. Patient characteristics were similar between groups except for the BID group having more patients who reported urinary frequency as a symptom (80.9% vs 69.3%, *P* = .035). Most patients received urinalysis testing in both groups. *E coli* was the primary pathogen identified, and the majority of patients received 7 days of therapy.

**Table 1.** Patient and Treatment Characteristics

	Median [IQR] or No. (%)		
Characteristic	BID (n = 173)	QID (n = 88)	*P* Value
Age, y, mean ± SD	55.4 ± 20.7	52.7 ± 20.4	.326
Weight, kg	72.7 [61.3–87.4]	76.7 [65.8–93]	.212
BMI, kg/m^2^	27.7 [23.9–33.1]	27 [24.3–34.6]	.472
CrCl, mL/min	87 [66.5–115]	100 [76–115]	.472
Diabetes	23 (13.3)	14 (15.9)	.567
Charlson Comorbidity Index	2 [0–3]	1 [0–4]	.468
Symptom			
Dysuria	135 (78)	68 (77.3)	.889
Increase urgency	109 (63)	47 (53.4)	.135
Increase frequency	140 (80.9)	61 (69.3)	.035
Suprapubic pain	49 (28.3)	30 (34.1)	.338
Other^a^	63 (36.4)	37 (43.2)	.289
Organism			.109
*Escherichia coli*	151 (87.3)	72 (81.8)	
*Klebsiella* spp	11 (6.4)	3 (3.4)	
*Proteus* spp	4 (2.3)	4 (4.5)	
*Staphylococcus saprophyticus*	5 (2.9)	2 (2.3)	
Other^b^	2 (1.1)	7 (8)	
Urinalysis obtained	168 (97.1)	84 (95.5)	.491
Duration of therapy			.241
5 d	36 (20.8)	24 (27.3)	
7 d	137 (79.2)	64 (72.7)	
Phenazopyridine prescribed	29 (16.8)	18 (20.5)	.463

Abbreviations: BID, twice daily; BMI, body mass index; CrCl, creatinine clearance; QID, 4 times daily.

a Hematuria, pelvic pain, incontinence, hesitancy, pressure, and incomplete emptying.

b *Aerococcus*, group B *Streptococcus*, *Citrobacter koseri*, and *Escherichia coli/Klebsiella* spp coisolated.

### Efficacy and Safety Outcomes

There was no difference in the primary outcome of treatment failure between groups (BID 12.7% vs QID 17%, *P* = .343), including failure while undergoing therapy or UTI recurrence within 30 days of initial therapy (Table 2). There was additionally no difference observed in median (IQR) days to treatment failure between groups (BID 13.5 [8–17] vs QID 10 [5–16], *P* = .276).

**Table 2.** Treatment Failure and Safety Outcomes

	No. (%) or Median [IQR]		
	BID (n = 173)	QID (n = 88)	*P* Value
Treatment failure	22 (12.7)	15 (17)	.343
During therapy	4 (2.3)	5 (5.7)	.438
Recurrence within 30 d	18 (10.4)	10 (11.3)	.438
Time to treatment failure, d	13.5 [8–17]	10 [5–16]	.276
Adverse event			.103
Nausea/vomiting/diarrhea	4 (2.3)	0 (0)	
Yeast infection	4 (2.3)	4 (4.5)	
Rash	0 (0)	1 (1.1)	
Hives	0 (0)	0 (0)	
Anaphylaxis	0 (0)	0 (0)	
*Clostridioides difficile* infection	0 (0)	0 (0)	>.99

Abbreviations: BID, twice daily; QID, 4 times daily.

Patient safety outcomes are displayed in Table 2. There were no differences in adverse events between groups (BID 4.6% vs QID 5.6%, *P* = .103). Yeast infections were the most common adverse event reported across both groups. There were no *Clostridioides difficile* infections within 30 days of treatment in either group (*P* > .99).

### Treatment Failure Outcomes

Of the 37 patients who experienced treatment failure (BID, n = 22; QID, n = 15), 16 (43.2%) had a new urine culture ordered within 30 days of completing cephalexin therapy (BID, n = 7; QID, n = 9), with 12 (32.4%) having a positive result (Table 3). *E coli* was the predominant pathogen isolated from reculture. One patient initially treated in the QID group had extended-spectrum β-lactamase *E coli*. The remaining *E coli* isolates were cephalexin susceptible. Cephalexin was the most common antibiotic prescribed for patients who experienced treatment failure in the BID group (36.4%), followed by nitrofurantoin (27.3%). Patients experiencing treatment failure in the QID group were most commonly treated with a fluoroquinolone (26.7%) or TMP/SMX (26.7%).

**Table 3.** Subgroup Analysis: Second Antibiotic/Pathogen for Treatment Failure

	No. (%)	
	BID	QID
Retreatment antibiotic	22	15
Cephalexin	8 (36.4)	3 (20)
Fluoroquinolone^a^	3 (13.6)	4 (26.7)
Fosfomycin	0 (0)	1 (6.7)
Nitrofurantoin	6 (27.3)	3 (20)
Trimethoprim/sulfamethoxazole	3 (13.6)	4 (26.7)
Unknown/undocumented^b^	2 (9.1)	0 (0)
Cultured pathogen	4	8
*Enterobacter* spp	0 (0)	1 (12.5)
*Escherichia coli*^c^	2 (50)	5 (62.5)
*Klebsiella* spp	1 (25)	0 (0)
*Proteus* spp	0 (0)	1 (12.5)
*Pseudomonas* spp	1 (25)	1 (12.5)

Abbreviations: BID, twice daily; QID, 4 times daily.

a Ciprofloxacin or levofloxacin.

b Unable to identify antibiotic prescribed due to limitations of electronic health record.

c One extended spectrum β-lactamase *Escherichia coli* found in QID group.

## DISCUSSION

Our findings demonstrate similar rates of treatment failure and time to failure in female patients receiving cephalexin 500 mg twice daily vs 4 times daily for uUTI. While historical data regarding uUTI treatment showed increased rates of failure with oral β-lactams and those administered for short courses, these studies were limited by the use of β-lactams with low oral bioavailability and urinary excretion as well as durations less than 5 to 7 days [2, 14, 15]. We observed a low rate of treatment failure (12.7%-17%) in both cephalexin treatment groups. This is similar to previous literature evaluating first-line therapies estimating that approximately 14% of uUTIs result in recurrence within 28 days of initial therapy [13]. β-Lactams are time-dependent antimicrobials; therefore, it is important to evaluate cephalexin's ability to achieve concentrations above the MIC in the urinary tract and to remain above the MIC for the majority of the dosing interval. The US Committee on Antimicrobial Susceptibility Testing and the Clinical and Laboratory Standards Institute currently recommend an MIC ≤16 mcg/mL as the breakpoint of cefazolin for predicting susceptibility to oral cephalosporin agents (eg, cephalexin) against Enterobacterales when treating infections within the urinary tract [12, 16]. A pharmacokinetic study by Harstein et al demonstrated that a single dose of cephalexin 500 mg yielded a urinary concentration of approximately 2400 mcg/mL, which is an estimated 150 times greater than the breakpoint [9]. The authors found that urinary concentrations remained above the MIC breakpoint for the entire 12-hour dosing interval, with a concentration of 49 mcg/mL reported 12 hours after the single 500-mg dose. These findings were corroborated in pharmacokinetic studies by Pfeffer et al and Welling et al evaluating urinary concentrations of cephalexin following a single 500-mg oral dose, further demonstrating cephalexin's potential as a potent agent in the treatment of uUTI [17, 18]. Oral cephalexin may represent an important empiric treatment option in the outpatient setting for health systems where ≥80% *E coli* urinary isolates demonstrate susceptibility to cefazolin [2]. Studies have shown that susceptibility reporting with inaccurate MIC breakpoints can lead to suboptimal prescribing and patient harm [19]. It is important to note the difference in cefazolin MIC breakpoints for urinary vs nonurinary Enterobacterales infections set by the Clinical and Laboratory Standards Institute; antimicrobial stewardship programs should ensure that MIC breakpoints for Enterobacterales urinary isolates are raised to ≤16 mcg/mL and that this is reflected on antibiogram reporting.

As the current national guidelines for the treatment of uUTI contain no recommended dosing for cephalexin, to our knowledge, there are minimal published data evaluating a cephalexin dosing strategy of 500 mg twice daily vs more frequent dosing. In 1983, Kostas et al compared cephalexin 500 mg twice daily and 250 mg dosed 4 times daily in 175 patients diagnosed with UTI [20]. The authors evaluated satisfactory symptomatic response within 5 to 9 days of antibiotic completion. Eighty-eight patients received the 500-mg twice-daily dosing strategy while 87 received 250 mg 4 times daily; the authors reported 94% and 93% satisfactory symptomatic responses, respectively, concluding that there was no difference in symptomatic cure between groups. Important limitations of this study include the short time to follow-up evaluation of symptomatic response, as well as an identical total daily cephalexin dose of 1 g being evaluated in both groups. Additionally, rates of Enterobacterales resistance have changed significantly since the time of publication. In 2023, Benning et al evaluated the feasibility of treating uUTI with cephalexin 500 mg twice daily in 264 patients discharged to home from the emergency department [21]. They reported clinical success at 30 days postdischarge in 81.1% of patients.

The authors importantly noted not only that their rate of clinical failure was due to nonresolving or worsening urinary symptoms but that the majority of failures were due to urine isolates indicated as nonsusceptible or inherently resistant to cephalexin. Additional limitations to note regarding the Benning et al study are the lack of a dosing strategy comparator group as well as the inclusion of patients who received doses of IV antibiotics and those who had no urine culture collected to determine their susceptibility to cephalexin. Our study strived to mitigate the limitations and potential confounders of these previously published clinical outcome studies by evaluating treatment failure up to 30 days following uUTI treatment and by excluding patients who received doses of IV antibiotics, had no urine culture collected, or had a urinary pathogen isolated that was not susceptible to cephalexin according to the current cefazolin breakpoint ≤16 mcg/mL. Additionally, our patient groups were well matched at baseline, including age, weight, and comorbidities such as diabetes.

The major benefit of reducing the frequency of cephalexin for uUTI is that it has the potential to improve patient adherence and satisfaction with treatment. Previous literature has shown that a reduction in medication doses needed throughout the day may lead to better adherence for patients with acute and chronic conditions and may improve quality of life and satisfaction with care [10, 11]. Additionally, reducing cephalexin dosing frequency and exposure may reduce the incidence of adverse events; however, we found that adverse drug reactions were low in both groups in our study. Provider preference of antimicrobial selection may also be affected by dosing frequency, with 4-times-daily regimens less favored. As uUTIs are one of the most common indications for outpatient antibiotic prescribing, provider education on optimizing cephalexin use through reducing dosing frequency is critical. Recommendations to reduce cephalexin dosing frequency to twice daily for uUTI may help improve utilization of cephalexin and limit prescribing of broader-spectrum oral second- and third-generation cephalosporins as well as less safe oral antimicrobial options, such as fluoroquinolones, which carry many safety warnings and are recommended to be avoided for uncomplicated infections [22]. At a time when resistance to first-line therapies and patient comorbidities are becoming more complex, cephalexin represents a safe and effective option for the treatment of uUTI for patients who are unable to safely or effectively receive treatment with nitrofurantoin or TMP/SMX.

There are important limitations to consider regarding our study. As with all retrospective analyses, there is a reliance on accurate documentation within the electronic medical record; this includes the reporting of urinary symptoms and adverse events via follow-up phone calls and documentation. We were also unable to assess patient adherence to the prescribed antimicrobial regimen; as concerns for adherence increase with the complexity of the regimen, it is possible that some patients in both groups may not have taken their full antibiotic course as prescribed. Additionally, the data collection of treatment failure and revisit or readmission relied on patients returning for care within our health system or other care facility connected to our health system's electronic health record. Therefore, if the patients did not have contact with the health care system within 30 days of completing therapy, they were assumed to have treatment success and no adverse events. Finally, our exclusion criteria were strict and may limit the application of our findings to certain populations, such as males, those with catheters, or patients with a history of frequent or recurrent UTIs. Additionally, we required patients to have a positive urine culture result for inclusion within the study to determine susceptibility; however, this is not considered the standard of care for all cases of uncomplicated cystitis. We hypothesize that patients who have urine cultures ordered for uUTI may be older with more comorbidities. Despite these strict exclusion criteria, we believe that our findings add important considerations for optimizing cephalexin dosing in female patients diagnosed with uUTI and support short durations of therapy (5–7 days) for this patient population.

## CONCLUSION

For female patients diagnosed with uUTI, cephalexin dosed 500 mg twice daily is equally effective as cephalexin dosed 500 mg 4 times per day. Twice-daily cephalexin dosing has the potential to improve patient adherence and satisfaction with the treatment course.

## Notes

***Author contributions.*** A. Y., H. M. D., K. E., and L. E. D. participated in study conception, design, data analysis and synthesis, and manuscript preparation and editing. A. P. J. participated in study design, data synthesis, and manuscript editing.

***Financial support.*** Financial support for the publication fees associated with this manuscript were provided by The Saint Mary's Foundation.
